# New Records of Canker-Causing Pathogens of *Acacia* spp. and *Pithecellobium dulce* in Southern Italy

**DOI:** 10.3390/jof11120874

**Published:** 2025-12-10

**Authors:** Giuseppa Rosaria Leonardi, Laura Vecchio, Giorgio Gusella, Dalia Aiello, Hermann Voglmayr, Giancarlo Polizzi

**Affiliations:** 1 Dipartimento di Agricoltura, Alimentazione e Ambiente (Di3A), Università degli Studi di Catania, Via S. Sofia 100, 95123 Catania, Italy; 2 Department of Botany and Biodiversity Research, University of Vienna, Rennweg 14, 1030 Vienna, Austria

**Keywords:** dieback, disease, fungal pathogens, phylogeny

## Abstract

Surveys conducted in a nursery located in eastern Sicily, southern Italy, revealed the presence of plants of *Vachellia nilotica* (syn. *Acacia arabica*), *V. farnesiana* (syn. *A. farnesiana*) and *Pithecellobium dulce* showing symptoms of trunk and branch canker, shoot dieback and general decline. Laboratory fungal isolation from wood tissues showed high percentage of *Diaporthe*-like (60–62%) and *Botryosphaeriaceae*-like fungi (21–85%) constantly associated with the diseased samples. Subsequent molecular characterization of recovered isolates was based on sequencing of the complete internally transcribed spacer region (ITS), the translation elongation factor 1-alpha (*tef1*) and the beta-tubulin (*tub2*) regions, followed by multi-locus phylogenetic analyses. The isolates collected from symptomatic tissues were phylogenetically characterized as *Diaporthe foeniculina* and *Neofusicoccum parvum*. Pathogenicity tests were conducted on *Acacia* and *P. dulce* plants and results showed that both species were pathogenic, being able to induce necrotic lesions on the stem. To our knowledge this is the first report worldwide of *D. foeniculina* and *N. parvum* infecting *A. arabica*, *A. farnesiana* and *P. dulce*.

## 1. Introduction

*Fabaceae* (or *Leguminosae*) [1] is an economically and ecologically important plant family including close to 770 genera and over 19,500 species [2]. This family includes *Acacia* species, some of them re-ordered nowadays within the genus *Vachellia*—woody perennial trees native to Australia, with some of them naturalized and invasive [3]—and *Pithecellobium dulce*, an evergreen plant native to Mexico known for its nutritional and medicinal properties [4]. In Italy, *Acacia* and *P. dulce* are cultivated and widely distributed, especially in the southern regions, for ornamental purposes. No relevant diseases have been reported for *P. dulce*; in fact, only few fungal associations have been listed, most of which with no symptoms recorded [5]. Regarding *Acacia* species, many fungal diseases have been reported worldwide, especially in tropical regions [6]. Since nurseries represent a key location in the production of plants, particular attention needs to be given to all strategies for preventing diseases occurring during this phase. In fact, diseases occurring in the nursery, especially those caused by canker-causing pathogens, are not always immediately detectable. Symptomatology can remain hidden throughout the latency of canker pathogens, and diseases may only be fully expressed after transplanting in the field [7].

Major fungal diseases of *Acacia* spp. occurring in the nursery include foliar diseases such as Pestalotiopsis leaf spot, Phaeotrichoconis leaf spot, phyllode rust disease (*Atelocauda digitata*) and anthracnose (*Colletotrichum* sp.), as well as stem and root diseases including seedling dumping-off caused by species belonging to *Pythium*, *Rhizoctonia* and *Fusarium* and various agents of canker and dieback [8].

Moreover, extensive literature is focused on the canker and wilt pathogen *Ceratocystis*, considered an emerging and important threat for *Acacia* plantations around the world [9,10,11,12,13]. As previously mentioned, in Italy, *Acacia* species are cultivated for ornamental purposes, which is the reason why the ornamental nurseries represent a crucial point for the detection of diseases that could compromise the propagation processes as well as the longevity of the plants in urban landscapes. In Italy, phytopathological investigations have not been particularly extensive. In this regard, the first disease detected in Italy was in 2001 on *A. retinoides*, when symptomatic plants showed leaf spot and stem canker, caused by *Calonectria pauciramosa* (reported as *Cylindrocladium pauciramosum*) [14]. In 2022, new symptoms of necrotic sunken lesions and wood discoloration were observed at the stem level in both the rootstock and the scion, as well as at the graft union of young plants of *A. dealbata* grafted on *A. retinodes* in a nursery in eastern Sicily. Pathogenicity test revealed *Lasiodiplodia citricola* as the causal agent of the disease [15].

The *Botryosphaeriaceae* and *Diaporthaceae* families include important canker pathogens of numerous agricultural, forestry and ornamental crops [16,17]. Symptomatology includes twig and shoot dieback, stem and trunk cankers, bark cracking, gummosis, and tree decline. These pathogens are often defined as opportunistic, able to survive as endophytes within the host tissues until the onset of stress conditions [16,17].

Recently, new surveys conducted in a nursery of eastern Sicily revealed the presence of plants of *Acacia arabica* (nowadays as *Vachellia nilotica*), *A. farnesiana* (*V. farnesiana*) and *P. dulce* showing symptoms of twig and shoot canker and dieback. For this reason, the aim of our study was to investigate the etiology of the disease by (i) characterizing the fungal isolates recovered from diseased wood samples based on phylogenetic analyses and (ii) testing their pathogenicity.

## 2. Materials and Methods

### 2.1. Surveys and Fungal Isolation

Surveys were carried out in a nursery located in the eastern area of Sicily during 2022. Symptomatic woody samples were collected from ten *Acacia* plants (five *A. arabica* plants and five *A. farnesiana* plants) and five *P. dulce* plants consisting of necrotized shoot, branch and trunk tissues. After collecting, samples were brought to the laboratory of the Department of Agriculture, Food and Environment, University of Catania, for further analyses. Fungal isolation was conducted as follows: small sections (0.2 to 0.3 cm^2^) of symptomatic woody tissues were surface-sterilized for 1 min in 1.5% sodium hypochlorite, rinsed in sterile deionized water, dried on sterile absorbent paper under a laminar hood, placed on potato dextrose agar (PDA, Lickson, Vicari, Italy) amended with 100 mg L^−1^ of streptomycin sulfate (Sigma-Aldrich, St. Louis, MO, USA) (PDA-S) to prevent bacterial growth, and then incubated at 25 °C for 3–5 days until fungal colonies were large enough to be examined. The isolation frequency (IF) of the main fungal categories was calculated according to the formula IF = (N_fs_/N_st_) × 100, where N_fs_ is the number of samples from which the specific fungal category was isolated and N_st_ is the total number of samples on which fungal isolation was conducted. Subsequently, fungi were grouped according to the general genus-family morphology of the colony (shape, color, texture) and representative colonies of interest were transferred to PDA-S plates. Single-hyphal tip cultures were obtained from pure cultures and maintained on PDA-S at 25 °C. From this preliminary grouping, representative isolates were chosen for molecular characterization.

### 2.2. DNA Extraction, PCR Amplification and Sequencing

Fungal isolates were grown on PDA for seven days for the genomic DNA extraction. Mycelium was collected and processed using the Wizard Genomic DNA Purification Kit^®^ (Promega Corporation, Madison, WI, USA) according to the manufacturer’s protocol. Extracted DNA was stored at 4 °C until use. The following gene regions were selected for amplification and sequencing: the complete internally transcribed spacer region (ITS1-5.8S-ITS2) rDNA gene region with primers ITS5 and ITS4 [18], the translation elongation factor 1-alpha (*tef1*) with primers EF1-728F and EF1-986R [19] and EF1-688F and EF1-1251R [20], and the beta-tubulin (*tub2*) region with primers Bt-2a and Bt-2b [21]. PCR conditions were set as follows: 30 s at 94 °C; 35 cycles each of 30 s at 94 °C; 1 min at 52 °C (ITS) or 55 °C (*tef1* and *tub2*); 1 min at 68 °C; and a final cycle for 5 min at 68 °C. PCR products were visualized on 1% agarose gels (90 V for 40 min) stained with GelRed^®^ Nucleic Acid GelStain (Biotium) to confirm the presence and size of PCR products. PCR amplicons were purified and sequenced in both direction by Macrogen Inc. (Seoul, Republic of Korea). The sequencing products were edited with Lasergene SeqMan Pro (DNASTAR, Madison, WI, USA) and deposited in GenBank (https://www.ncbi.nlm.nih.gov/). Isolates characterized in this study are listed in Table 1.

### 2.3. Phylogenetic Analysis

The sequences obtained in this study were compared with the NCBI GenBank nucleotide database using the standard nucleotide Basic Local Alignment Search Tool (BLAST) (https://blast.ncbi.nlm.nih.gov/Blast.cgi (accessed on 7 May 2024)). The newly generated sequences of each genomic region were aligned to reference sequences retrieved from recent and comprehensive phylogenetic studies for isolates in the genera *Diaporthe* [22] and *Neofusicoccum* [23] and downloaded from GenBank (Table 2). Sequence alignments for phylogenetic analyses were produced with the server version of MAFFT (https://mafft.cbrc.jp/alignment/server/ (accessed on 10 June 2025)) and checked and refined using BioEdit Sequence alignment Editor 7.7.1.0 [24]. Isolates in both genera were used for phylogenetic analyses within a combined matrix of ITS rDNA, *tef1* and *tub2* sequences. The loci were concatenated to a combined matrix using Phyutility v. 2.2 [25] (Smith and Dunn 2008). Sequences of *Botryosphaeria dothidea* and *B. fabicerciana* served as outgroup taxa in phylogenetic analyses of the family *Botryosphaeriaceae*, and *Diaporthella corylina* served as the outgroup taxon in phylogenetic analyses of the family *Diaporthaceae*. Maximum likelihood (ML) analyses were performed with RAxML [26], as implemented in raxmlGUI 2.0 [27], using the ML + rapid bootstrap setting and the GTRGAMMA+I substitution model which was selected as the most appropriate model by Modeltest. The matrix was partitioned for the different gene regions, and bootstrap analyses were performed with 1000 bootstrap replicates. For evaluation and interpretation of bootstrap support, values between 70% and 90% were considered moderate, above 90% as high, and 100% as the maximum. Maximum parsimony (MP) bootstrap analyses were performed with Phylogenetic Analyses Using Parsimony (PAUP) v. 4.0a169 [28]. A total of 1000 bootstrap replicates were implemented using five rounds of heuristic search with random sequence addition, followed by tree-bisection-reconnection (TBR) branch swapping. The MULTREES option was enabled, the steepest descent option was disabled, the COLLAPSE command was set to MINBRLEN, and each replicate was limited to 1 million rearrangements. All molecular characters were treated as unordered and assigned equal weight, with gaps considered as missing data. The COLLAPSE command was set to MINBRLEN.

### 2.4. Pathogenicity Test

Two species of *Acacia*, including *A. arabica* and *A. farnesiana*, and the species *P. dulce* were selected to conduct pathogenicity tests in order to fulfill Koch’s postulates. Regarding *A. arabica* and *A. farnesiana*, a total of six plants for each plant species were inoculated with the fungal isolates. Specifically, three plants were inoculated with *D. foeniculina* isolate ACA 91, and three plants with *N. parvum* isolate ACA 82. Likewise, three plants of *P. dulce* were inoculated with *D. foeniculina* isolate ACA 113 and three with *N. parvum* ACA 105. Three plants served as controls. Stem wounds were made with a sterilized 5 mm diameter cork borer to remove the bark, and a 5 mm diameter mycelium plug from a seven-day-old culture of the selected isolates was placed upside down into the wound. Wounds were then sealed with Parafilm^®^ to prevent desiccation. Controls were inoculated with sterile PDA plugs. Plants were regularly watered. Total lesion lengths were measured 60 days after inoculation. Re-isolations were conducted as described above and identification was based on morphological characteristics (color, texture, growth rate and eventually spore features) of the colonies.

## 3. Results

### 3.1. Surveys and Fungal Isolation

**Figure 1:**
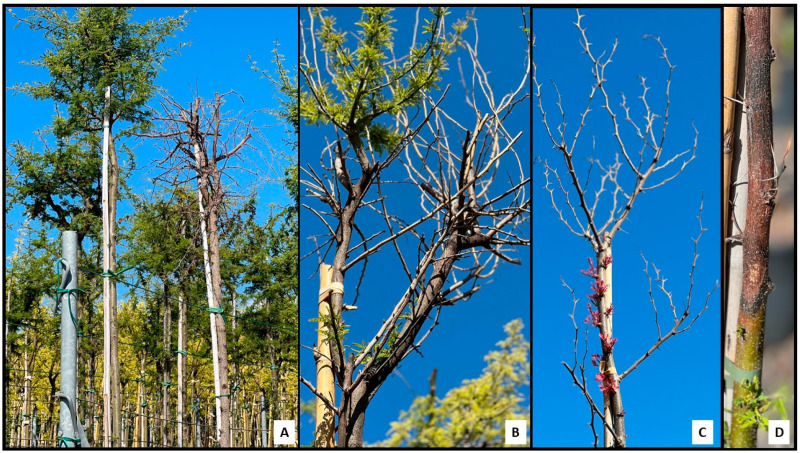
Symptomatology of *Acacia* spp. and *Pithecellobium dulce*. (**A**), *Acacia* spp. plant decline. (**B**), apical shoot dieback on *Acacia* plant. (**C**), *P. dulce* dieback and canopy defoliation. (**D**), stem canker and necrosis on *Acacia* plant.

Disease incidence observed in the nursery was about 9% based on a total of 2000 cultivated plants. Specifically, about 6% was observed for *Acacia* plants and 3% for *P. dulce* plants. The symptomatology on *Acacia* spp. and *P. dulce* consisted of typical apical shoot dieback and general decline of the plants (Figure 1A–D), as well as necrotic patches and external and internal necrotic lesions along the trunks and shoots and at the insertion of the main branches. The isolation frequency from *Acacia* plants consisted of 62% of *Diaporthe*-like colonies from dieback symptoms and 21% of *Botryosphaeriaceae*-like colonies from necrotic lesions on trunks and branches. For *P. dulce* plants, the isolation frequency showed 85% of *Botryosphaeriaceae*-like colonies from necrotic lesions on trunks and branches and 60% of *Diaporthe*-like from twig dieback. From fungal isolation, a total of 46 isolates (32 *Diaporthe*-like and 14 *Neofusicoccum*-like) were collected and stored in the fungal collection of the Department of Agriculture, Food and Environment, Laboratory of Plant Pathology. The isolates collected belonging to each genus (*Diaporthe* and *Neofusicoccum*) did not show any morphological differences. Thus, a total of 31 representative isolates (23 *Diaporthe*-like and 8 *Neofusicoccum*-like) were selected for further molecular analyses.

### 3.2. Phylogenetic Analysis

**Figure 2:**
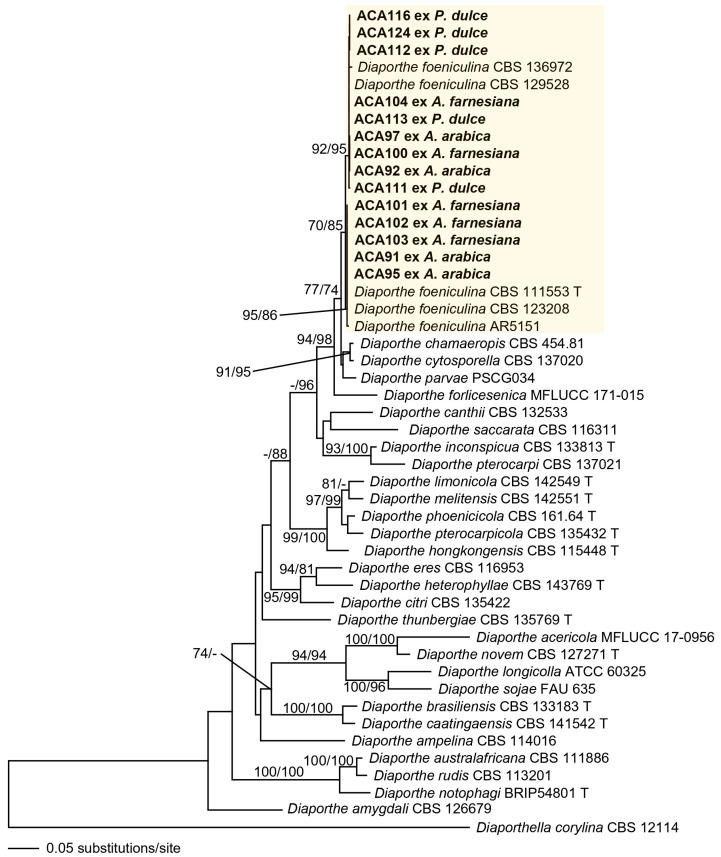
Phylogram of the best ML tree (−lnL = 12,314.431617) revealed by RAxML from an analysis of the combined ITS-*tef1-tub2* matrix of *Diaporthe*, showing the phylogenetic position of isolates from diseased *Acacia arabica*, *A. farnesiana* and *Pithecellobium dulce* plants (bold), with *Diaporthella corylina* (CBS 12114) selected as outgroup to root the tree. Maximum likelihood (ML) and maximum parsimony (MP) bootstrap support above 70% are given at first and second position, respectively, above or below the branches. T = ex-type.

Since the *Diaporthe* isolates revealed identical sequences for the analyzed loci (ITS, *tef1*, *tub2*), except for some ITS polymorphisms, 14 representative isolates were selected for the phylogenetic analyses. The dataset used for the phylogenetic analyses of *Diaporthe* consisted of 48 taxa, including the isolates from *Acacia* spp. and *P. dulce* and the outgroup (*D. corylina* CBS 121124). The combined matrix of ITS-*tef1*-*tub2* included 1610 characters (597 ITS, 428 *tef1*, 585 *tub2*), of which 871 were constant (399 ITS, 132 *tef1*, 340 *tub2*), 233 were variable but parsimony-uninformative (85 ITS, 62 *tef1*, 86 *tub2*) and 506 were parsimony informative (113 ITS, 134 *tef1*, 159 *tub2*). The ML tree (−lnL = 12,314.431617) obtained by RAxML is shown in Figure 2. The isolates ACA 91, ACA92, ACA 95, ACA97, ACA100-104, ACA 111-113, ACA 116 and ACA124 were collected from the symptomatic plants clustered with *D. foeniculina* with medium support (70% ML, 85% MP). However, two strongly supported lineages were distinguished within this clade, suggesting an intraspecific variability within *D. foeniculina* isolates.

**Figure 3:**
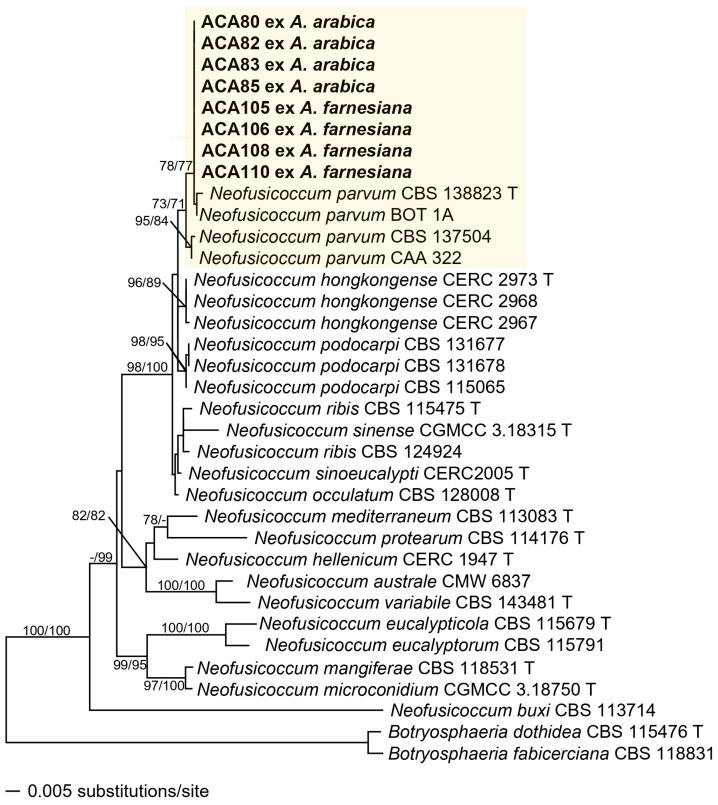
Phylogram of the best ML tree (−lnL = 3893.049301) revealed by RAxML from an analysis of the combined ITS-*tef1-tub2* matrix of *Neofusicoccum*, showing the phylogenetic position of isolates from diseased *Acacia arabica*, *A. farnesiana* and *Pithecellobium dulce* plants (bold), with *Botryosphaeria dothidea* (CBS 115476) and *B. fabicerciana* (CBS 118831) selected as outgroup to root the tree. Maximum likelihood (ML) and maximum parsimony (MP) bootstrap support above 70% are given at first and second position, respectively, above or below the branches. T = ex-type.

The dataset of *Neofusicoccum* contained 40 taxa including the isolates from *Acacia* spp. and *P. dulce* and the outgroups (*Botryosphaeria dothidea* CBS 115476 and *B. fabicerciana* CBS 118831). The alignments included 1255 characters (534 ITS, 306 *tef1*, 415 *tub2*) of which 989 were constant (450 ITS, 196 *tef1*, 343 *tub2*), 84 were variable but parsimony-uninformative (29 ITS, 30 *tef1*, 25 *tub2*) and 182 parsimony informative (55 ITS, 80 *tef1*, 47 *tub2*). The ML tree (−lnL = 3893.049301) obtained by RAxML is shown in Figure 3. Maximum likelihood analyses resulted in a tree topology similar to that revealed by MP bootstrap analysis. Phylogenetic analyses did not show intraspecific variability among the ACA isolates, which were all resolved inside the *N. parvum* s. str. clade with moderate (73% ML, 71% MP) support. However, they were strongly supported (98% ML, 100% MP) within the *N. parvum* species complex, which includes *N. hongkongense*, *N. occulatum*, *N. parvum*, *N. podocarpi*, *N. ribis* and *N. sinoeucalypti*.

### 3.3. Pathogenicity Test

**Figure 4:**
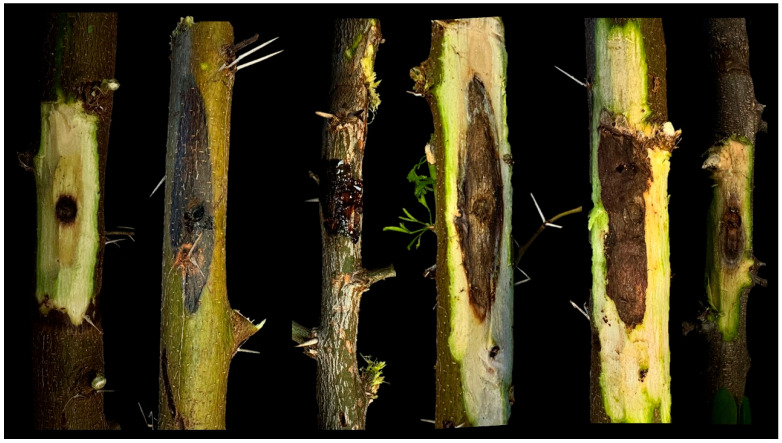
Pathogenicity test. From left to right: non-inoculated control plant; external lesion caused by *Diaporthe foeniculina* isolate ACA 91 on *Acacia arabica;* gummosis starting from inoculation point of *D. foeniculina* isolate ACA 91 on *Acacia farnesiana*; internal lesion caused by *D. foeniculina* isolate ACA 91 on *Acacia arabica*; internal lesion on *Acacia arabica* inoculated with *Neofusicoccum parvum* isolate ACA 82.

Results of pathogenicity test proved that both fungal species are pathogenic to *Acacia* plants as well as to *P. dulce* although with some slight differences (Figure 4). In particular, *D. foeniculina* isolate ACA 91 and *N. parvum* isolate ACA 82 induced lesions on *A. arabica* with an average length of 5.6 (standard deviation ± 2.4) cm and 5.1 (±2.2) cm, respectively. On *A. farnesiana*, *D. foeniculina* isolate ACA 91 induced lesions of an average of 7.5 (±4.7) cm, and *N. parvum* isolate ACA 82 of 5.7 (±4.1) cm. On *P. dulce*, *D. foeniculina* isolate ACA 113 and *N. parvum* isolate ACA 105 induced lesions of an average of 1.9 ± 1.0 cm and 1.9 ± 0.3 cm, respectively. Of note, gum production was observed from the inoculation point of *A. farnesiana* plants. Control plants did not produce any lesions, but a superficial discoloration that did not extend beyond the inoculation point was observed due to the oxidation of wounded tissue. Re-isolations from all inoculated plants confirmed the presence of the *Diaporthe-* and *Neofusiccocum*-like colonies, with cultural characteristics matching those of the inoculated isolates. No colonies resembling *Diaporthe* or *Neofusicoccum* were isolated from control plants.

## 4. Discussion

The results of this study revealed the presence of *D. foeniculina* and *N. parvum*, causing cankers and dieback, on *A. arabica*, *A. farnesiana* and *P. dulce* in a nursery located in Sicily, southern Italy. These plant species are cultivated in Italy mainly for ornamental purposes, and some *Acacia* spp. are also recognized as alien and invasive species. For example, *A. saligna* was introduced for reforestation purposes and for dune stabilization, but it quickly became an invasive species across the entire national territory [29]. The attention to *Acacia* is testified in Italy by reforestation programs conducted during the 1950s–1960s in southern Italy with the species *A. melanoxylon* for its appreciable wood and botanical characteristic in preventing the spread of wildfire [30].

Wood diseases are increasingly becoming the subject of investigation of plant pathologists around the world due to the increasing complexity of their etiology and their wide host range and challenging management [31]. In our study, *N. parvum* (*Botryosphaeriaceae*) was consistently isolated from symptomatic tissues of *A. arabica* and *A. farnesiana*. This species is widely distributed around the world and well known to be a highly aggressive canker-causing pathogen on many different crops, including ornamental trees [32,33]. Its wide distribution, probably a result of the repeated introductions of agricultural and ornamental plant material [34], and its ability to attack many different hosts, characteristic of several *Botryosphaeriaceae* [17], make the report of this pathogen significant for the growers. In Sicily, *N. parvum* has been repeatedly reported in recent decades, demonstrating its highly polyphagous behavior for ornamental [35,36,37,38] and agricultural crops [39,40,41]. This species, with identification based only on the ITS gene region, was reported in Sicily on *A. melanoxylon* causing canker and dieback in 2016 [30]. However, for a proper identification of *Neofusicoccum* species, a multi-gene phylogenetic analysis is necessary, especially in the case of *N. parvum*, which is part of a cryptic species complex [23].

Similarly, the presence of *D. foeniculina* on both *Acacia* species is not unusual. *Diaporthaceae* are recognized as another important group of fungi causing wood diseases on several fruit and nut crops [31], and many associations of *Diaporthe* species with *Acacia* have been recorded [42]. Although *D. foeniculina* is reported worldwide as a primary canker-causing pathogen [43,44], some studies considered it as a weak pathogen, less aggressive compared to other fungal species [41,45,46,47]. On the contrary, in our study, lesions on *A. arabica* and *A. farnesiana* caused by *D. foeniculina* are similar, in terms of length, to those produced by *N. parvum*. Thus, results of our study do not suggest a clear demarcation between the two identified species in disease development. Discrepancies regarding the observed aggressiveness of the same fungal species around the world may be quite normal and can be attributed to different factors such as differences in isolate virulence and host response.

Concerning *P. dulce*, this study provides the first report of cankers and dieback caused by *D. foeniculina* and *N. parvum*. Until now, only a few diseases have been reported on this host, of which none seemed to be relevant in terms of limitation to its cultivation [5]. The results of our investigation highlight the need to not underestimate the risks derived by the development of wood diseases, especially in nurseries. Canker-causing pathogens, in fact, are characterized by phases of latency during the infection cycle [17] that make it almost impossible to ascertain their presence within the host, unless molecular techniques such as qPCR [48] are used. Latent infections of canker-causing pathogens establishing without notice in the nursery represent the initial inoculum from which further epidemics could develop in new fields [7], especially when plants are subjected to different types of stress like injuries, heat and drought [17]. This epidemiological statement was also evidenced, for example, in the cases of apple canker caused by *Nectria galligena*, *Botryosphaeriaceae* diseases on almond and prune, and grapevine infected by several wood pathogens [7,49,50]. In this regard, especially in nurseries and greenhouses, it is crucial to maintain high standards of hygiene during all delicate processes of propagation. Routine sanitation does not guarantee the complete absence of inoculum in plants but could be very helpful in keeping the potential inoculum under control. Monitoring of pathogen populations in diversified environments, like nurseries, where many plant species co-inhabit is crucial to avoid dangerous host jumps and intensification of disease levels.

To our knowledge, this is the first report of *D. foeniculina* and *N. parvum* causing canker and dieback on *Acacia* species and *P. dulce* worldwide, and these species also should be monitored in other areas where their hosts are planted to evaluate their spread and impact.

## Figures and Tables

**Table 1:** Isolates collected from symptomatic *Acacia* spp. and *Pithecellobium dulce* plants used in the molecular analyses.

Species	Strain ^a^	Host	Country	GenBank Accession Number ^b^		
				ITS	*tef1*	*tub2*
*Diaporthe foeniculina*	**ACA 91**	*Acacia arabica*	Italy, Giarre	PX649194	PX662033	PX662010
*Diaporthe foeniculina*	**ACA 92**	*Acacia arabica*	Italy, Giarre	PX644738	PX662034	PX662011
*Diaporthe foeniculina*	**ACA 95**	*Acacia arabica*	Italy, Giarre	PX649195	PX662035	PX662012
*Diaporthe foeniculina*	ACA 96	*Acacia arabica*	Italy, Giarre	PX644739	PX662036	PX662013
*Diaporthe foeniculina*	**ACA 97**	*Acacia arabica*	Italy, Giarre	PX644740	PX662037	PX662014
*Diaporthe foeniculina*	ACA 98	*Acacia arabica*	Italy, Giarre	PX644741	PX662038	PX662015
*Diaporthe foeniculina*	ACA 99	*Acacia farnesiana*	Italy, Giarre	PX644742	PX662039	PX662016
*Diaporthe foeniculina*	**ACA 100**	*Acacia farnesiana*	Italy, Giarre	PX644743	PX662040	PX662017
*Diaporthe foeniculina*	**ACA 101**	*Acacia farnesiana*	Italy, Giarre	PX649196	PX662041	PX662018
*Diaporthe foeniculina*	**ACA 102**	*Acacia farnesiana*	Italy, Giarre	PX649197	PX662042	PX662019
*Diaporthe foeniculina*	**ACA 103**	*Acacia farnesiana*	Italy, Giarre	PX649198	PX662043	PX662020
*Diaporthe foeniculina*	**ACA 104**	*Acacia farnesiana*	Italy, Giarre	PX644744	PX662044	PX662021
*Diaporthe foeniculina*	**ACA 111**	*Pithecellobium dulce*	Italy, Giarre	PX644745	PX662045	PX662022
*Diaporthe foeniculina*	**ACA 112**	*Pithecellobium dulce*	Italy, Giarre	PX644746	PX662046	PX662023
*Diaporthe foeniculina*	**ACA 113**	*Pithecellobium dulce*	Italy, Giarre	PX644747	PX662047	PX662024
*Diaporthe foeniculina*	**ACA 116**	*Pithecellobium dulce*	Italy, Giarre	PX644748	PX662048	PX662025
*Diaporthe foeniculina*	ACA 117	*Pithecellobium dulce*	Italy, Giarre	PX649199	PX662049	PX662026
*Diaporthe foeniculina*	ACA 118	*Pithecellobium dulce*	Italy, Giarre	PX649200	PX662050	PX662027
*Diaporthe foeniculina*	ACA 119	*Pithecellobium dulce*	Italy, Giarre	PX644749	PX662051	PX662028
*Diaporthe foeniculina*	ACA 122	*Pithecellobium dulce*	Italy, Giarre	PX644750	PX662052	PX662029
*Diaporthe foeniculina*	ACA 123	*Pithecellobium dulce*	Italy, Giarre	PX649201	PX662053	PX662030
*Diaporthe foeniculina*	**ACA 124**	*Pithecellobium dulce*	Italy, Giarre	PX644751	PX662054	PX662031
*Diaporthe foeniculina*	ACA 126	*Pithecellobium dulce*	Italy, Giarre	PX644752	PX662055	PX662032
*Neofusicoccum parvum*	**ACA 80**	*Acacia arabica*	Italy, Giarre	PX651383	PX662056	PX662064
*Neofusicoccum parvum*	**ACA 82**	*Acacia arabica*	Italy, Giarre	PX651384	PX662057	PX662065
*Neofusicoccum parvum*	**ACA 83**	*Acacia arabica*	Italy, Giarre	PX651385	PX662058	PX662066
*Neofusicoccum parvum*	**ACA 85**	*Acacia farnesiana*	Italy, Giarre	PX651386	PX662059	PX662067
*Neofusicoccum parvum*	**ACA 105**	*Pithecellobium dulce*	Italy, Giarre	PX651387	PX662060	PX662068
*Neofusicoccum parvum*	**ACA 106**	*Pithecellobium dulce*	Italy, Giarre	PX651388	PX662061	PX662069
*Neofusicoccum parvum*	**ACA 108**	*Pithecellobium dulce*	Italy, Giarre	PX651389	PX662062	PX662070
*Neofusicoccum parvum*	**ACA 110**	*Pithecellobium dulce*	Italy, Giarre	PX651390	PX662063	PX662071

^a^ Isolates and sequences used in the phylogenetic analyses are shown in bold font; ^b^ ITS, internal transcribed spacer; *tef1*, translation elongation factor 1-α; *tub2*, beta-tubulin.

**Table 2:** GenBank accession numbers of isolates used in the phylogenetic analyses.

Species	Strain ^a^	Host	Country	GenBank Accession Number ^b^		
				ITS	*tef1*	*tub2*
*Botryosphaeria dothidea*	CBS 115476 T	*Prunus* sp.	Switzerland	AY236949	AY236898	AY236927
*Botryosphaeria fabicerciana*	CBS 118831	*Syzygium cordatum*	South Africa	DQ316084	MT592032	MT592468
*Diaporthe acericola*	MFLUCC 17-0956	*Acer negundo*	Italy	KY964224	KY964180	KY964074
*Diaporthe ampelina*	CBS 114016	*Vitis vinifera*	France	AF230751	GQ250351	JX275452
*Diaporthe amygdali*	CBS 126679	*Prunus dulcis*	Portugal	KC343022	KC343748	KC343990
*Diaporthe australafricana*	CBS 111886	*Vitis vinifera*	Australia	KC343038	KC343764	KC344006
*Diaporthe brasiliensis*	CBS 133183 T	*Aspidosperma tomentosus*	Brazil	KC343042	KC343768	KC344010
*Diaporthe caatingaensis*	CBS 141542 T	*Tacinga inamoena*	Brazil	KY085926	KY115603	KY115600
*Diaporthe chamaeropis*	CBS 454.81	*Chamaerops humilis*	Greece	KC343048	KC343774	KC344016
*Diaporthe canthii*	CBS 132533	*Canthium inerme*	South Africa	JX069864	KC843120	KC843230
*Diaporthe citri*	CBS 135422	*Citrus* sp.	USA	KC843311	KC843071	KC843187
*Diaporthe cytosporella*	CBS 137020	*Citrus limon*	Spain	KC843307	KC843116	KC843221
*Diaporthe eres*	CBS 116953	*Pyrus pyrifolia*	New Zealand	KC343147	KC343873	KC344115
*Diaporthe foeniculina*	CBS 111553 T	*Foeniculum vulgare*	Portugal	KC343101	KC343827	KC344069
*Diaporthe foeniculina*	CBS 129528	*Rhus pendulina*	South Africa	JF951146	KC843100	KC843205
*Diaporthe foeniculina*	CBS 123208	*Foeniculum vulgare*	Portugal	EU814480	GQ250315	JX275464
*Diaporthe foeniculina*	CBS 136972	*Vaccinium corymbosum*	Italy	KJ160565	KJ160597	MF418509
*Diaporthe foeniculina*	AR5151	*Citrus latifolia*	USA	KC843303	KC843112	KC843217
*Diaporthe forlicesenica*	MFLUCC 17-1015	*Dorycnium hirsutum*	Italy	KY964215	KY964171	KY964099
*Diaporthe hongkongensis*	CBS 115448 T	*Dichroa febrifuga*	China	KC343119	KC343845	KC344087
*Diaporthe heterophyllae*	CBS 143769 T	*Acacia heterophylla*	France	MG600222	MG600224	MG600226
*Diaporthe inconspicua*	CBS 133813 T	*Maytenus ilicifolia*	Brazil	KC343123	KC343849	KC344091
*Diaporthe limonicola*	CBS 142549 T	*Citrus limon*	Malta	MF418422	MF418501	MF418582
*Diaporthe longicolla*	ATCC 60325	*Glycine max*	USA	KJ590728	KJ590767	KJ610883
*Diaporthe melitensis*	CBS 142551 T	*Citrus limon*	Malta	MF418424	MF418503	MF418584
*Diaporthe notophagi*	BRIP54801 T	*Notophagus cunninghamii*	Australia	JX862530	JX862536	KF170922
*Diaporthe novem*	CBS 127271 T	*Glycine max*	Croatia	KC343156	KC343882	KC344124
*Diaporthe parvae*	PSCG034	*Pyrus bretschneideri*	China	MK626919	MK654858	MK691248
*Diaporthe phoenicicola*	CBS 161.64 T	*Areca catechu*	India	KC343032	KC343758	KC344000
*Diaporthe pterocarpi*	CBS 137021	*Pterocarpus indicus*	Thailand	JQ619901	JX275418	JX275462
*Diaporthe pterocarpicola*	CBS 135432 T	*Pterocarpus indicus*	Thailand	JQ619887	JX275403	JX275441
*Diaporthe rudis*	CBS 113201	*Vits vinifera*	Portugal	KC343234	KC343960	KC344202
*Diaporthe sojae*	FAU 635	*Glycine max*	USA	KJ590719	KJ590762	KJ610875
*Diaporthe saccarata*	CBS 116311	*Protea repens*	South Africa	KC343190	KC343916	KC344158
*Diaporthe thunbergiae*	CBS 135769 T	*Thunbergia laurifolia*	Thailand	JQ619893	JX275409	JX275449
*Diaporthella corylina*	CBS 12114 T	*Corylus* sp.	China	KC343004	KC343730	KC343972
*Neofusicoccum buxi*	CBS 113714	*Buxus sempervirens*	Sweden	KX464164	KX464677	KX464954
*Neofusicoccum australe*	CMW 6837	*Acacia* sp.	Australia	AY339262	AY339270	AY339254
*Neofusicoccum eucalypticola*	CBS 115679 T	*Eucalyptus grandis*	Australia	AY615141	AY615133	AY615125
*Neofusicoccum eucalyptorum*	CBS 115791	*Eucalyptus grandis*	South Africa	AF283686	AY236891	AY236920
*Neofusicoccum hellenicum*	CERC 1947 T	*Pistacia vera*	Greece	KP217053	KP217061	KP217069
*Neofusicoccum hongkongense*	CERC 2973 T	*Araucaria cunninghamii*	China	KX278052	KX278157	KX278261
*Neofusicoccum hongkongense*	CERC 2967	*Araucaria cunninghamii*	China	KX278050	KX278155	KX278259
*Neofusicoccum hongkongense*	CERC 2968	*Araucaria cunninghamii*	China	KX278051	KX278156	KX278260
*Neofusicoccum mangiferae*	CBS 118531 T	*Mangifera indica*	Australia	AY615185	DQ093221	AY615172
*Neofusicoccum mediterraneum*	CBS 113083 T	*Pistacia vera*	U.S.A.	KX464186	KX464712	KX464998
*Neofusicoccum microconidium*	CGMCC 3.18750 T	*Eucalyptus urophylla* × *E. grandis*	China	KX278053	KX278158	KX278262
*Neofusicoccum occulatum*	CBS 128008 T	*Eucalyptus grandis*	Australia	EU301030	EU339509	EU339472
*Neofusicoccum parvum*	CBS 138823 T	*Populus nigra*	New Zealand	AY236943	AY236888	AY236917
*Neofusicoccum parvum*	CBS 137504	*Vitis vinifera*	Algeria	KJ657702	KJ657715	KX505915
*Neofusicoccum parvum*	CBS 140887	*Vitis vinifera* cv. Alfrocheiro	Portugal	MT587448	MT592158	MT592648
*Neofusicoccum parvum*	CBS 139672	*Bruguiera gymnorhiza*	South Africa	MT587446	MT592156	MT592646
*Neofusicoccum parvum*	BOT 1A	*Citrus limon*	Italy	MW727244	MW789904	MW789889
*Neofusicoccum parvum*	CAA 322	*Malus domestica*	Portugal	KX505906	KX505894	KX505916
*Neofusicoccum parvum*	CPC 32275	*Pomaderris aspera*	Australia	MT587451	MT592160	MT592651
*Neofusicoccum parvum*	CPC 35861	*Aloe* sp.	South Africa	MT587449	MT592159	MT592649
*Neofusicoccum parvum*	CBS 139671	*Bruguiera gymnorhiza*	South Africa	MT587445	MT592155	MT592645
*Neofusicoccum podocarpi*	CBS 131677	*Podocarpus henkelii*	South Africa	MT587508	MT592223	MT592715
*Neofusicoccum podocarpi*	CBS 115065	*Wollemia nobilis*	Australia	MT587507	MT592222	MT592714
*Neofusicoccum podocarpi*	CBS 131678	*Podocarpus henkelii*	South Africa	MT587509	MT592224	MT592716
*Neofusicoccum protearum*	CBS 114176 T	*Leucadendron salignum*	South Africa	AF452539	KX464720	KX465006
*Neofusicoccum ribis*	CBS 115475 T	*Ribes* sp.	USA	AY236935	AY236877	AY236906
*Neofusicoccum ribis*	CBS 124924	*Terminalia catappa*	Cameroon	FJ900607	FJ900653	FJ900634
*Neofusicoccum sinense*	CGMCC 3.18315 T	Unknown woody plant	China	KY350148	KY817755	KY350154
*Neofusicoccum sinoeucalypti*	CERC2005 T	*Eucalyptus urophylla* × *E. grandis*	China	KX278061	KX278166	KX278270
*Neofusicoccum variabile*	CBS 143481 T	*Mimusops caffra*	South Africa	MH558610	MH576586	MH569155

^a^ T = ex-type isolates. ^b^ ITS, internal transcribed spacer; *tef1*, translation elongation factor 1-α; *tub2*, beta-tubulin.

## Data Availability

The original data presented in the study are openly available in GenBank (https://www.ncbi.nlm.nih.gov/genbank).
